# Estimating the causal effect of annual PM_2.5_ exposure on mortality rates in the Northeastern and mid-Atlantic states

**DOI:** 10.1097/EE9.0000000000000052

**Published:** 2019-06-17

**Authors:** Maayan Yitshak-Sade, Itai Kloog, Antonella Zanobetti, Joel D. Schwartz

**Affiliations:** a Exposure, Epidemiology, and Risk Program, Department of Environmental Health, Harvard T.H. Chan School of Public Health, Boston, Massachusetts; b Department of Geography and Environmental Development, Faculty of Humanities and Social Sciences, Ben-Gurion University, Beer-Sheva, Israel; c Channing Division of Network Medicine, Department of Medicine, Brigham and Women’s Hospital and Harvard Medical School, Boston, Massachusetts.

**Keywords:** Causal modeling, PM2.5, Mortality, Air pollution

## Abstract

**Background:**

Dozens of cohort studies have associated particulate matter smaller than 2.5 µm in diameter (PM_2.5_) exposure with early deaths, and the Global Burden of Disease identified PM_2.5_ as the fifth-ranking mortality risk factor in 2015. However, few studies have used causal modeling techniques. We assessed the effect of annual PM_2.5_ exposure on all-cause mortality rates among the Medicare population in the Northeastern and mid-Atlantic states, using the difference-in-differences approach for causal modeling.

**Methods:**

We obtained records of Medicare beneficiaries 65 years of age or more who reside in the Northeastern or mid-Atlantic states from 2000 to 2013 and followed each participant from the year of enrollment to the last year of follow-up. We estimated the causal effect of annual PM_2.5_ exposure on mortality rates using the difference-in-differences approach in the Poisson survival analysis. We controlled for individual confounders, for spatial differences using dummy variables for each ZIP code and for time trends using a penalized spline of year.

**Results:**

We included 112,376,805 person-years from 15,401,064 people, of whom 37.4% died during the study period. The interquartile range (IQR) of the annual PM_2.5_ concentration was 3 µg/m^3^, and the mean annual PM_2.5_ concentration ranged between 6.5 and 14.5 µg/m^3^ during the study period. An IQR incremental increase in PM_2.5_ was associated with a 4.04% increase (95% CI = 3.49%, 4.59%) in mortality rates.

**Conclusions:**

Assuming no omitted predictors changing differently across ZIP codes over time in correlation with PM_2.5_, we found a causal effect of PM_2.5_ on mortality incidence rate.

**What this study adds**

Numerous studies found higher mortality risks associated with long-term exposure to particulate matter smaller than 2.5 µm in diameter (PM_2.5_). However, because randomized controlled trials are not feasible when studying population-based air pollution effects, the majority of the evidence originates from observational approaches. This cohort study of 112,376,805 person-years shows a significant effect of PM_2.5_ on the risk of mortality among the elderly population. Therefore, the associations between PM_2.5_ and mortality remain when using a casual modeling approach designed to overcome the limitations of a nonrandomized observational study.

## Introduction

Numerous epidemiological studies have concluded that long-term exposure to particulate matter smaller than 2.5 µm in diameter (PM_2.5_) increases the risk of mortality, shortens life expectancy, and increases the risk of cardiovascular and respiratory morbidity.^1–13^ The Global Burden of Diseases, Injuries, and Risk Factors study ranked air pollution as the fifth highest mortality risk factor in 2015, mostly from ischemic heart disease and cerebrovascular disease.^1^

Randomized controlled trials (RCTs) are not feasible when studying population-based air pollution effects. Therefore, the majority of the evidence relies on classical observational approaches. Unlike RCTs, where the randomization assures that the exposure of interest is independent of all other parameters at the time of randomization, classical observational approaches are prone to confounding bias.^14^ The causal modeling approach overcomes this limitation by using methods designed to mimic the randomization process as much as possible.

Causal modeling methods follow the potential outcome approach and aim to estimate the difference between the outcome of an exposed population, and the expected outcome of the same population had they received different exposure. Because that counterfactual outcome is missing by definition, causal modeling methods seek unbiased ways to estimate these unobserved potential outcomes.^15–17^

Studies that used these causal modeling methods for air pollution have become more common in recent years,^15,18,19^ but are still scarce. A common approach is the use of propensity score matching or inverse probability weighting, to make the exposure of interest independent of all measured confounders.^15,16^ Makar et al^18^ used a propensity score approach in a representative sample of 32,119 Medicare beneficiaries. Wang et al^19^ applied a doubly robust causal modeling estimate with inverse probability weights to look at PM_2.5_ and mortality in the Southeastern United States. The disadvantage of these methods is that it can only account for measured confounders and for unmeasured confounders that are highly correlated with the measured confounders.^20^

The difference-in-differences (DID) approach overcomes this limitation and allows controlling for unmeasured confounders by design. In recent decades, the increasing availability of routinely collected electronic health records has provided the opportunity to conduct large population-based studies. However, because the data are not collected for specific study aims, many potential confounders remain unmeasured and may bias the studied associations. Hence, the importance of controlling for these unmeasured confounders.^21^

In a classical DID model, mean outcomes are calculated for the exposed and nonexposed groups in the preexposure and postexposure periods. The difference between preexposure and postexposure periods in the unexposed group is a negative outcome control for the difference in the exposed group, and the difference in these pairs of differences is a causal estimate, assuming that no other exposure has affected the two groups differently over time.^22^ This is because the difference between death rates in the same location between two periods controls for all slowly varying predictors of mortality such as socioeconomic status (SES), smoking, obesity, etc. The difference between the two periods in the control location controls for time trends in an outcome that is similar between the two locations.

In a previous analysis done by our group, Wang et al^23^ applied a variant of the DID approach among all mortality cases in New Jersey. We applied the DID approach of Wang et al^23^ to the Medicare population of the Northeastern and mid-Atlantic states, extended the analysis to incorporate individual covariates, and assessed the effect of annual PM_2.5_ exposure on all-cause mortality rates.

## Methods

### Study population

We included all the Medicare beneficiaries 65 years of age or more who reside in the following states from 2000 to 2013: Maine, New Hampshire, Vermont, Massachusetts, Rhode Island, Connecticut, New York, New Jersey, Delaware, Pennsylvania, Maryland, Washington, D.C., Virginia, and West Virginia. Participants entered the cohort on January 1 of the year after they became Medicare participants, and were followed for each calendar year until death or end of the study. This study was approved by the human subjects committee at the Harvard T. H. Chan School of Public Health.

### Exposure data

Our group generated highly spatially resolved PM_2.5_ data (1 × 1 km spatial resolution) from a hybrid satellite-based model incorporating daily satellite remote sensing Aerosol Optic Depth data and classic land-use regression methodologies. We used out-of-sample 10-fold cross-validation to quantify the accuracy of the model predictions and found excellent model performance for both days with available satellite data (mean out-of-sample *R*^2^ = 0.88) and days without satellite observations (mean out-of-sample *R*^2^ = 0.87). For more in-depth description, please refer to Kloog et al.^24^

We averaged the exposure values of all grid cells located within each ZIP code and a 500 meters buffer annually and assigned exposure data to ZIP codes using ArcGIS based on spatial location.

### Covariates

The Medicare denominator file includes data on the participant’s age, sex, race, date of death, ZIP code of residence, and eligibility for Medicaid, which provides additional coverage for low-income participants.

We obtained the mean annual temperature in the summer and winter seasons from the North American Regional Reanalysis data and estimated daily mean values for each ZIP code in our study area.^25^

### Statistical analysis

We used a causal modeling approach to assess the effect of long-term PM_2.5_ exposure on the rate of death. Specifically, we used the DID approach in a Poisson survival analysis using the Anderson–Gill formulation with time-varying covariates.^26^ We have extended the classical DID approach to continuous exposures and multiple time periods.^23^ We used a similar approach in this study, but added some controls for individual covariates. We followed Medicare participants in 7600 ZIP codes in the Northeastern and mid-Atlantic states. We begin with the potential outcomes framework of the Rubin Causal Model.^27^ Let *Y*^*A*=*a*^_*i,c,t*_ be the potential outcome in person I, in ZIP code *c* if exposed to *A = a* in year *t*, and let *Y*^*A=a*^*′*_*i,c,t*_ be the potential outcome under the alternative exposure *A = a*′. We assumed that the incidence of death depends on predictors in the following manner:


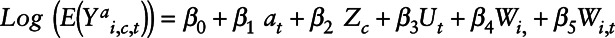
(1)

where *Z*_*c*_ is small area level confounders that vary between ZIP codes but not over the study period; *U*_*t*_ is temporal confounders that vary over time but not between ZIP codes; *W*_*i*_ is the person’s individual confounders that do not vary over time and *W*_*i,t*_ is the person’s individual confounders that do vary over time.

A dummy variable for each ZIP code will effectively remove confounding by variables that only vary across exposure groups (*Z*_*c*_ and *W*_*i*_). Similarly, a penalized spline for the year of study will remove all the temporal confounders that vary between periods (*U*_*t*_ and *W*_*i,t*_). The adjustment to the individual characteristics will account for individual confounders that may not be captured by adjustment to the ZIP code of residence. Then, the remaining variability of the annual PM_2.5_ levels can be treated as the difference in these pairs of differences (i.e., the causal estimate). The benefit of this approach is that we are able to control for unmeasured confounders, because they are removed by the time trend and ZIP code intercepts. This is a generalization of the original DID approach where there were only two treatment units and two time periods, and the causal effect of treatment was the interaction term. It reduces to the same thing in that case.^23,28^

## The DID assumptions

For a causal interpretation of the DID estimate, the following assumptions must hold^29^:

1. The intervention (or the exposure, in this case) is unrelated to the outcome at baseline. Meaning that the level of annual PM_2.5_ exposure is not determined by the mortality rates.
2. All the ZIP codes have parallel trends in mortality rates. Meaning that, had the PM_2.5_ levels not been changing the same over time, the difference in mortality rates between the ZIP codes will be stable over time as well. This can be weakened to allow differences in the trend in mortality rates over time by ZIP code due to other factors, as long as those differences are not correlated with differences in PM_2.5_ over time by ZIP code.

For the first assumption to hold, mortality in a current year should not affect the current exposure and should not be affected by past exposures. In a classical DID approach, there is a defined driver for the change in exposure between the preintervention and postintervention periods (usually an intervention), which can potentially be affected by the outcome. This seems unlikely in this case, because EPA policy changes are not impacted by Northeast mortality rates.

Regarding the second assumption, we must assume that differences from year to year in *W*_*i,t*_ which are not common across ZIP codes are not correlated with PM_2.5_. That is, as long as the annual *within* ZIP code changes in individual smoking rates, diet, etc., which are different from the same changes elsewhere are not correlated with annual *within* ZIP code changes in PM_2.5_ that are different from the same changes elsewhere, then we will obtain a causal estimate of the effect of PM_2.5_. Because this assumption cannot be tested statistically, it must be judged based on knowledge and plausibility from outside the study. The only factors that may potentially confound the association are factors like smoking rates or SES that may vary differently with time across ZIP codes. However, for these variables to confound the association, it requires a highly unlikely scenario in which these variations are correlated with variations in PM_2.5_ levels from the average ZIP code level and the average annual level.

The long-term trend in exposure is removed by the penalized spline, and the exposure contrast is the local deviation from that trend. That can be parsed into two parts: difference between the long-term trend in the ZIP code from the trend in the Northeastern United States and fluctuations about that trend from year to year. The major driver of trends in PM_2.5_ in the Northeast in this time period was EPA required reductions in NOx and SO_2_ emissions at upwind power plants. ZIP code trends in PM_2.5_ concentrations will vary depending on the extent to which the ZIP codes are downwind of the plants that put on controls during the period, and when the controls were installed. All these factors are unlikely related to trends in SES, smoking, or any other potential confounder.^23^ That said, as an additional measure to control for potential confounding, we adjusted the models for individual characteristics that may be related with SES. The remaining variability in PM_2.5_ depends mostly on meteorological factors, such as year-to-year differences in mixing height and wind speed, which seem random with respect to year-to-year differences from trend in ZIP code mortality rates.

However, it is possible that mortality in the current year will be related to exposures in earlier years. We, therefore, added two sensitivity analyses aimed to make sure that the effects of current year exposure are not confounded by earlier exposures. First, we repeated our model, assessing the effect of PM_2.5_ in the year of death (time *t*), with adjustment for PM_2.5_ exposure in the previous year (time *t*−1). Second, we obtained the residuals from our main analysis, which assessed the effect of PM_2.5_ in time *t* on mortality, adjusting for time trends and ZIP code differences. We then assessed the mean within ZIP code correlation between the residuals in time *t* and time *t*−1.

The one variable that has the potential to confound the tested association despite the adjustment for the spatial, temporal, and individual confounders is temperature. We, therefore, adjusted the models to seasonal temperatures.

**Figure 1.**
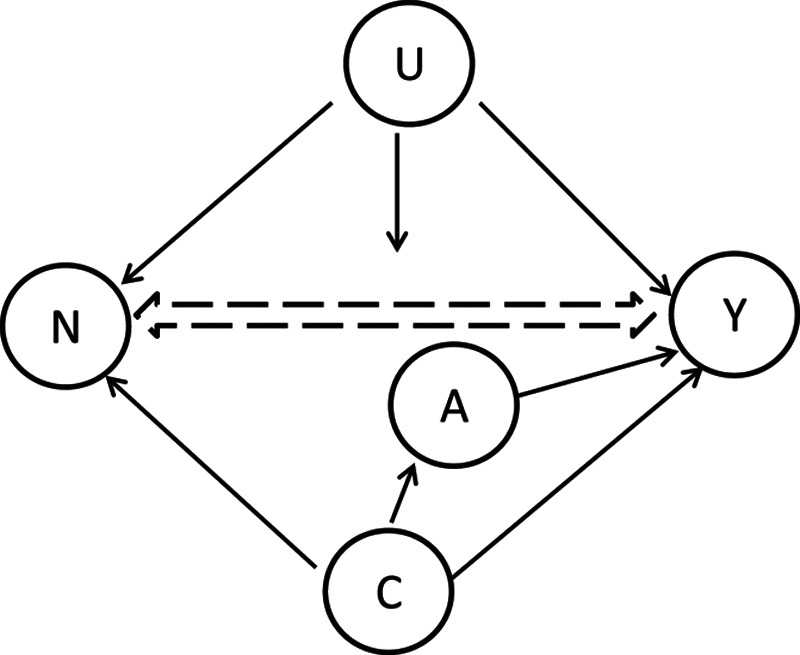
Directed acyclic graph for the causal association between air pollution exposure (A), mortality as the primary outcome (Y), fatty liver disease as the negative outcome control (N), measured confounders (C), and unmeasured confounders (U).

To support our findings, we added a sensitivity analysis analyzing the association between PM_2.5_ exposure and alcoholic fatty liver disease, as a negative outcome control. This condition is a result of alcohol consumption, and therefore, theoretically should not be related to PM_2.5_ exposure. Therefore, an association would suggest residual confounding by unmeasured confounders as shown in the Directed acyclic graph (Figure 1).

To further address the possibility of confounding by differential changes in SES across ZIP codes, we conducted an additional sensitivity analysis controlling for annual estimates of population density, percent over 65 years of age and living in poverty, and median household income.

Due to computational limitations arising from the large sample size, we randomly split the data into subsets and applied our models on each subset. The effect estimates were then pooled using a fixed-effect meta-analysis. Results are presented as percent change for IQR increase in annual exposure.

We assessed modification of the PM_2.5_ and mortality association by adding multiplicative interaction terms with race and eligibility to Medicaid services. Each interaction term was assessed separately.

## Results

**Figure 2.**
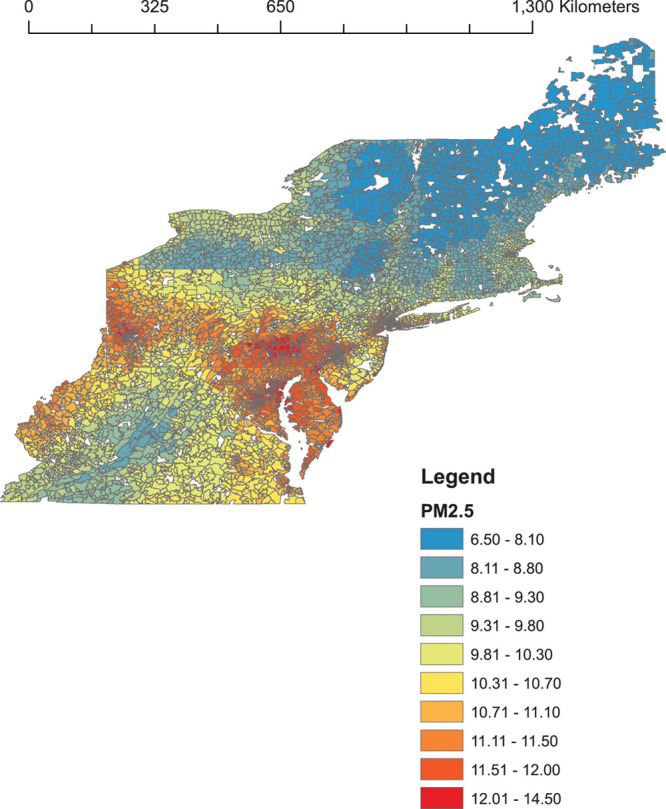
The ZIP codes included in the study and the centiles of the mean annual PM_2.5_.

We included 112,376,805 person-years from 15,401,064 people, of whom 37.4% have died during the study period. The mean age of the study population was 78 years, and the mean age of death was 82 years, 42.8% were males, 85.2% were white, and 16.2% had dual eligibility for the Medicare and Medicaid services (Table 1). The IQR of the annual PM_2.5_ concentration was 3 µg/m^3^. Figure 2 shows the states and ZIP codes included in the study and the centiles of the mean annual PM_2.5_ concentration, ranging between 6.5 and 14.5 µg/m^3^ throughout the study period (Figure 2).

**Table 1:**
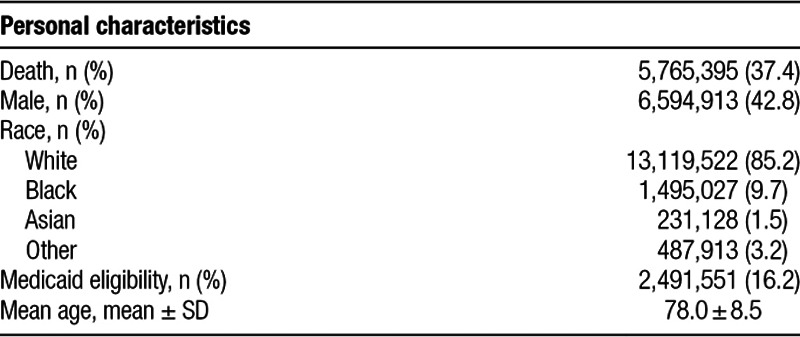
Population characteristics (n = 15,401,064 people)

After adjustment for personal characteristics and the spatial and temporal annual variation in PM_2.5_, an IQR incremental increase in PM_2.5_ was associated with a 4.04% increase (95% CI = 3.49%, 4.59%; *P* < 0.001) in mortality rates. The effect was modified by eligibility to Medicaid insurance and race (interaction *P* value <0.001 for both), with larger effects among people who are eligible to Medicaid Services (5.99%; 95% CI = 4.38%, 7.62%) and among blacks (10.10%; 95% CI = 8.56%, 11.67%) (Table 2).

**Table 2:**
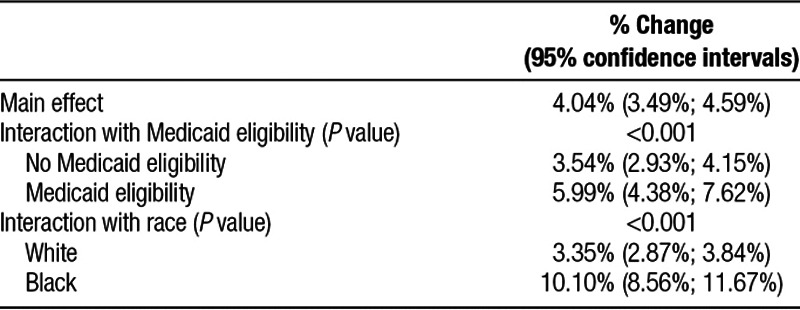
Percent increase in total mortality, associated with interquartile range increase (3 µg/m^3^) in annual PM_2.5_ exposure

We conducted several sensitivity analyses to assess whether the assumptions, required for a causal interpretation of the estimate, are met. First, when we repeated our model with adjustment to the PM_2.5_ exposure in the previous year. The effect of PM_2.5_ in the current year remained unchanged (4.10% increase; 95% CI = 3.55%, 4.65%; *P* < 0.001), and we observed no association with the earlier exposure (0.33% increase; 95% CI = −0.19%, 0.86%; *P* = 0.189). In addition, after removing the variability in the outcome that is related to PM_2.5_ in the current year, ZIP code differences, time trend, and individual confounders – the mean Pearson’s correlation – of the within ZIP code residuals, was very low (*r* = −0.02). Second, when we tested the association with alcoholic fatty liver as a negative outcome, we found no association with PM_2.5_ (3.0% increase; 95% CI = −4.7%, 11.4%; *P* = 0.456). Third, when we added adjustment for annual estimates of percent of persons 65 years of age and more living in poverty, population density, and median household income, the effect was similar (3.88%; 95% CI = 3.33%, 4.43%) to the base analysis. In addition, the correlation between median income in the first and last year of the study by ZIP code was 0.86, suggesting that there has been little trend in status by ZIP code during the period.

## Discussion

This study of 14 years of follow-up involved all Medicare beneficiaries of 14 US states, includes a large number of persons and deaths, and also captures participants who reside in rural areas. We found a significant effect of PM_2.5_ on the risk of mortality using a method that controls for many, and possibly all, omitted confounders. These findings suggest that the associations between PM_2.5_ and mortality remain when using a casual modeling approach designed to overcome the limitations of a nonrandomized observational study. The specific approach used in this study only examines the association of year-to-year changes in PM_2.5_ within ZIP code with year-to-year changes in mortality risk within ZIP code, after removing time trends from both, and control for some key individual covariates.

These results are in line with results from another study conducted among this study population, which estimated the distribution of life expectancy as a function of annual PM_2.5_ exposure. Comparing the percent of the population that would die before the age of 76 years, the authors found higher mortality rates among those exposed to PM_2.5_ at 12 µg/m^3^ (23.5%) than those exposed to PM_2.5_ at 7 µg/m^3^ (20.1%). While this study uses a different methodology and a different outcome, it support our findings of the causal effect of PM_2.5_ on mortality.^30^

We believe the trends within ZIP code exposures found in our study are likely to be independent of potential confounders, and therefore, the association identified is likely causal. It is possible that events occurred during the study period that affected both the exposure and the outcome differentially across ZIP code, and therefore may still confound the association. For example, if a major industrial site was closed, this has the potential to affect both PM_2.5_ levels and mortality rate only among ZIP code which are located closer to the factory. However, because our analysis only included the elderly population, this scenario is less likely. Furthermore, such a change would likely be felt through a change in SES, and control for annual SES in each ZIP code did not modify our results. In addition, since most of the change in PM_2.5_ in our study period is due to nonlocal sources, we think that differential temporal changes by zip codes and other predictors of health are not likely to be correlated. The decline in PM_2.5_ in the United States over this time period derives from two basic sources. First, SO_2_, and NOx controls were retrofit on many coal-burning power plants during this time period. This reduced concentrations of sulfate, nitrate, and secondary organic aerosols downwind of those plants and the impact was felt primarily in ZIP codes hundreds of miles downwind. However, because that assignment to more or less secondary particles is random with respect to socioeconomic changes, or other predictors of health, and is rather the result of wind patterns, we do not expect it to confound the association. The second source of decline was the imposition of particle controls on new Diesel engines, and the gradual retirement of older trucks. Because this reduction in emissions is due to lower emissions rates per truck, and not less truck traffic, it is less likely to impact socioeconomic patterns.

Sofer et al^31^ suggested the negative outcome controls can be used to detect confounding bias. A negative outcome can be any measured outcome that is not causally related to the exposure of interest and is influenced by the same unmeasured confounders of the exposure–outcome association of interest.^31^ We chose alcoholic fatty liver as a negative outcome and found no association with PM_2.5_. This suggests that unmeasured confounders do not confound the association of interest in our study.

The association between particulate pollution, morbidity, and mortality has been well documented in many observational studies.^8,11,32–35^ Even with the decreasing trend of air pollution, PM_2.5_ exposure is still linked to excess mortality.^32^ For example, a recent cohort study of all Medicare beneficiaries in the United States found a 7.3% increase in all-cause mortality associated with 10 µg/m^3^ increase in annual PM_2.5_ exposure.^12^ Another example is a study that included seven US southeastern states and found a 23% increase in all-cause mortality associated with 10 µg/m^3^ increase in annual PM_2.5_ exposure.^36^ These associations, reported repeatedly in many settings, different populations, and geographical areas are compelling. However, by definition, these studies describe associations and do not prove causation.

Controlled exposure studies can assess underlying mechanisms and prove causation.^37^ A few controlled exposure studies that assessed the effect of air pollution on ECG changes^38^ or heart rate variability^39^ were conducted over the years. Another trial has randomized people to either functional or sham in home particle filters for a year and documented a 7–mm Hg difference in blood pressure.^40^ These trials can provide indirect support for the effect of air pollution exposure on mortality by identifying mechanisms. Despite the important advantage of randomization and controlled exposure, the major limitation of these studies is the ethical issues that arise when exposing individuals to these toxic components.

Animal toxicology studies can also provide mechanistic support for a conclusion of causality. For example, mice on a high-fat diet exposed for 6 months to an average particle concentrations of 15.2 μg/m^3^ had almost twice the atherosclerotic plaque as animals exposed to filtered air.^41^ Another study reported that lung function in mice exposed to outdoor air, with average particle concentrations of 16.8 μg/m^3^, was lower than in mice exposed to filtered air, with particle concentrations of 2.9 μg/m^3^.^42^

The use of causal modeling techniques allowed us to conduct a large-scale population-based study while obtaining an estimate for the PM_2.5_ effect that is causal if our assumptions are met. Similar to our study, a previous analysis done by our group Wang and colleagues^23^ has estimated the causal effects of long-term exposure to PM_2.5_ on mortality. The authors found 3% increase in all-cause mortality, associated with an IQR increase (2 µg/m^3^) in annual PM_2.5_ exposure, among mortality cases in New Jersey.^23^

Another study that estimated the causal effect of 2-year exposure to PM_2.5_ on mortality among a representative sample of Medicare beneficiaries found that increases in PM_2.5_ were associated with increases of all-cause and cause-specific hospital admission rates but did not find evidence of an increase in mortality.^18^ However, that study had only a one-year follow-up on 32,119 individuals and the number of deaths in that follow-up period was small. The different inferences may be due to the use of individual survival data, the size of the cohort and the number of deaths analyzed, the selected population, and the adjustment for personal confounders.

In addition to the methods and study design, another term required for the determination of a causal effect is biological plausibility. Two possible mechanisms that link PM_2.5_ exposure with mortality are commonly described in the literature. First, the development of pulmonary oxidative stress may lead to respiratory mortality or an indirect effect on the circulatory system. The later may cause cardiovascular events, arrhythmia, and even death as a result of the inflammatory responses, vascular dysfunction, and the accumulation of cytokines and clotting factors. Second, ultrafine particles can penetrate directly into the circulatory system through the pulmonary system and cause local oxidative stress, which may lead to the same cardiovascular and mortality outcomes.^32,43^

We found larger effects among people with eligibility for Medicaid services and blacks. People dual eligibility to Medicaid are often of lower SES.^23,44,45^ The larger effects found in these populations may be related to the higher likelihood of non-white and low-income populations to be exposed to higher pollution levels.^44,46^ It can also be related to limited financial and physical access to healthcare services.^44,47–49^

The large sample size and geographical area covered in this study are the major strengths of the study. Using a satellite-based model to estimate PM_2.5_ exposure, we were able to include both urban and rural population and to obtain exposure in high spatial resolution. In addition, if our assumption that all the time-varying confounders were accounted for in the models, holds, we were able to identify the causal association between PM_2.5_ and mortality.

Our study also has limitations. First, we were not able to directly adjust the models to predictors such as comorbidities, smoking or BMI. To the extent that those predictors varied slowly during the follow-up period, or similarly within ZIP codes, they were removed by the DID approach. We believe any remaining variation in such predictors will be uncorrelated with within ZIP code year-to-year variations in PM_2.5_, but that is an untestable hypothesis. An examination of a random sample of the Medicare cohort that did have individual-level information on such covariates found no association between them and PM_2.5_ even before using the DID approach.^12^ Trying to minimize confounding by individual factors, we did control for the measured personal and socioeconomic predictors. Second, despite the use of highly spatially resolved exposure models, because we assigned the same annual average to each ZIP code, exposure misclassification may still be present. However, because the potential error is Berksonian, it may increase the confidence intervals but should not bias the estimates.

In conclusion, assuming no predictors changing differentially across ZIP codes over time other than those accounted for in our model, we found a causal effect of PM_2.5_ on the mortality incidence rate. The negative outcome control approach provides farther strength to our findings.

## Acknowledgments

The authors declare that they have no conflicts of interest with regard to the content of this report.
